# Spatial and Temporal Shifts in Bacterial Biogeography and Gland Occupation during the Development of a Chronic Infection

**DOI:** 10.1128/mBio.01705-16

**Published:** 2016-10-11

**Authors:** Daniela Keilberg, Yana Zavros, Benjamin Shepherd, Nina R. Salama, Karen M. Ottemann

**Affiliations:** a Department of Microbiology and Toxicology, University of California Santa Cruz, Santa Cruz, California, USA; b Department of Molecular and Cellular Physiology, University of Cincinnati, Cincinnati, Ohio, USA; c Division of Human Biology, Fred Hutchinson Cancer Research Center, Seattle, Washington, USA

## Abstract

Gland colonization may be one crucial route for bacteria to maintain chronic gastrointestinal infection. We developed a quantitative gland isolation method to allow robust bacterial population analysis and applied it to the gastric pathobiont *Helicobacter pylori*. After infections in the murine model system, *H. pylori* populations multiply both inside and outside glands in a manner that requires the bacteria to be motile and chemotactic. *H. pylori* is able to achieve gland densities averaging 25 to 40 bacteria/gland after 2 to 4 weeks of infection. After 2 to 4 weeks of infection, a primary infection leads to colonization resistance for a secondary infection. Nonetheless, about ~50% of the glands remained unoccupied, suggesting there are as-yet unappreciated parameters that prevent gastric gland colonization. During chronic infections, *H. pylori* populations collapsed to nearly exclusive gland localization, to an average of <8 bacteria/gland, and only 10% of glands occupied. We analyzed an *H. pylori* chemotaxis mutant (Che^−^) to gain mechanistic insight into gland colonization. Che^−^ strains had a severe inability to spread to new glands and did not protect from a secondary infection but nonetheless achieved a chronic gland colonization state numerically similar to that of the wild type. Overall, our analysis shows that bacteria undergo substantial population dynamics on the route to chronic colonization, that bacterial gland populations are maintained at a low level during chronic infection, and that established gland populations inhibit subsequent colonization. Understanding the parameters that promote chronic colonization will allow the future successful design of beneficial microbial therapeutics that are able to maintain long-term mammalian colonization.

## IMPORTANCE

Many bacteria have an impressive ability to stay in the gastrointestinal tract for decades despite ongoing flow and antimicrobial attacks. How this staying power is achieved is not fully understood, but it is important to understand as scientists plan so-called designer microbiomes. The gastrointestinal tract is lined with repeated invaginations called glands, which may provide one niche for chronic colonization. We developed a quantitative gland isolation method to allow robust and efficient bacterial population analysis and applied it to the gastric pathogen *Helicobacter pylori*. Bacterial populations increased inside and outside glands at early time points but were found exclusively within glands during late time points in the chronic state. *H. pylori* required the ability to swim to move to new glands. Last, a fit gland bacterial population leads to colonization resistance of a second one. Our approach identified previously unappreciated aspects of gland occupation, supporting the idea that glands are the desired niche for stable, chronic colonization.

## INTRODUCTION

Studies of the microbial habitats of the human body have revealed that there are distinct stable communities covering skin, as well as the respiratory, gastrointestinal, and urogenital tracts (1). The mammalian gastrointestinal tract is home to thousands of bacterial species that are able to maintain chronic colonization (2). Different bacterial species compete for colonization and resources, and ultimately influence the abundance of each other (2). A long-term goal of microbiology research is to be able to engineer the colonization of specific microbial flora to create a so-called designer intestinal microbiome and in turn influence human health (3, 4). To be able to achieve this goal, we need a strong understanding of the molecular mechanisms that drive chronic colonization inside the gastrointestinal tract.

The gastrointestinal epithelial tissue is extensively invaginated, creating numerous pockets that are called glands in the stomach, and crypts in the intestine and colon. Evidence from intestinal colonization models with *Bacteroides* spp. suggests that bacterial colonization deep within these glands is one route to maintain long-term colonization (5). Several pathogenic bacterial species have been shown to reside within glands, including *Helicobacter pylori*, *Campylobacter jejuni*, *Vibrio cholerae*, *Escherichia coli*, and *Shigella flexneri* (6–12). However, the importance of the gland populations to disease or sustained colonization is not yet known. Part of the challenge for gland analysis has been that the typical approach used—tissue sectioning and microscopy—is time-consuming, and analyses are limited to small numbers of glands and a small portion of the tissue (7, 10–12). This limitation has prevented a detailed quantitative understanding of factors and mechanisms required for gland colonization, leaving gaps in our understanding of the kinetics and bacterial distribution inside and outside the glands.

One microbe that has been shown to colonize gastrointestinal glands is the pathobiont *H. pylori*. *H. pylori* infection is typically acquired in childhood and becomes chronic, lasting for the life of the host (13–15). *H. pylori* infection has a range of outcomes from asymptomatic gastritis to gastric ulcers to cancer (16, 17). *H. pylori* infection additionally can modulate the host immune response to protect against diseases such as asthma (16). How *H. pylori* maintains chronic infection is not well understood.

Previous studies have identified two bacterial factors that are required for gland or crypt colonization. The first identified was chemotactic motility in *H. pylori* (7). Specifically, *H. pylori* mutants lacking chemotaxis (Che^−^) were found less frequently in gastric glands (7, 11). In *Bacteroides fragilis*, a nonpathogenic bacterium, specific sugar utilization pathways were found to be required to allow gland colonization (5). Sugar utilization and chemotaxis share the function of promoting nutrient acquisition, so one hypothesis is that gland colonization requires the ability to locate and utilize specific nutrients.

Chemotaxis is a process by which bacteria sense their environment and move toward beneficial compounds and away from harmful ones. *H. pylori* senses several chemotaxis signals, including the surrounding pH, urea, amino acids, autoinducer 2, and metals via one of four chemoreceptor sensing proteins (18–23). Chemoreceptors in turn control a histidine kinase signal transduction system that phosphorylates CheY (24, 25). The ratio of CheY to CheY-phosphate modulates the direction of flagellar rotation via interaction with the flagellar motor and determines whether the bacteria swim forward or reverse. Inactivation of any of the crucial proteins along this signal cascade, including CheY, lead to *H. pylori* bacteria that are unable to control their swimming and have substantial stomach colonization defects (23, 26–28).

In this work, we quantify *H. pylori* gland colonization kinetics by employing a new method for isolating and analyzing glands from infected mice that we call bacterial localization in isolated glands (BLIG). The BLIG method allows an efficient quantitative analysis of bacterial numbers inside and outside the glands, over time, and in distinct anatomical portions of the stomach. We find that gland colonization starts at the beginning of infection and proceeds at different rates in different portions of the stomach. Bacterial populations increase both inside and outside glands, suggesting that both niches support *H. pylori* growth. At the height of infection, the infected glands average 25 to 40 bacteria/gland, but the stomach saturates at ~50% of glands, leaving ~50% of the glands uncolonized even in the face of repeated high-level *H. pylori* challenge. A stomach colonized by wild-type bacteria can display colonization resistance, however, against colonization by a second strain. In contrast to the early phase of acute infection, during long-term chronic infection, the bacterial population undergoes a severe restriction to become exclusively gland localized, with low numbers of bacteria per gland and few glands infected. We applied our analysis method to determine that chemotaxis is required for early high-level gland colonization but not to achieve the low-level chronic state. Overall, our analytical methods enable a high-resolution picture of how bacterial populations change over time in the mammalian gastrointestinal tract.

## RESULTS

### *H. pylori* gland colonization can be readily quantified *ex vivo* in isolated glands.

**FIG 1:**
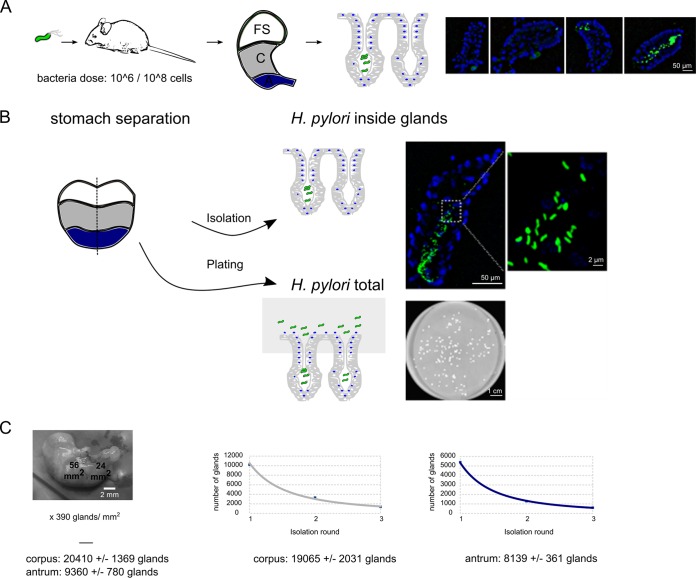
Methodology for gland isolation and quantification of bacteria and glands (A) The BLIG gland isolation method. (Left) The mice were infected with fluorescently labeled *H. pylori* strains. (Middle) The stomachs were isolated and separated (fore stomach [FS], corpus [C], and antrum). (Right) Glands with bacteria inside were isolated. Strain SS1 was chosen because it colonizes to high levels and is genetically stable over the course of infection. Bacteria were visualized by GFP expression (green), while gland cells were visualized using a DNA Hoechst stain (blue). Isolated glands displaying a range of different bacterial colonization levels are shown in the series of photographs, which show from left to right, empty glands to low and high colonization. (B) Overall protocol in which stomachs were isolated from infected mice and separated into two corpus pieces and two antrum pieces. One piece from the corpus and one piece from the antrum were homogenized and plated to obtain the total CFUs; the second pieces were used for BLIG gland isolation. Bacterial numbers were determined using microscopy. (C) Glands in the corpus and antrum were quantified by two methods. (Left) The area of antrum and corpus tissue of mouse stomachs was measured, and the total number of glands was calculated using the previously determined gland density of 390 glands/mm^2^ (35), multiplied by the area. (Right) The glands were isolated from antrum and corpus tissue in three sequential rounds, and the total numbers of glands were extrapolated from the decreasing gland numbers counted in the isolation process.

To enable analysis of microbial gland colonization, we developed a protocol in which mice were first infected with *H. pylori* strains that expressed green or red fluorescent protein (GFP or RFP, respectively), and then glands were separated from the rest of the stomach tissue (Fig. 1). We termed this approach bacterial localization in isolated glands (BLIG). Glands were isolated using a well-established protocol that is used as the first step in the generation of gastric organoids (29–31) and gives rise to a high density of isolated glands that retain normal gland morphology. For all work, we used an *H. pylori* strain that has been optimized for mouse infection and does not appear to undergo as frequent genetic changes during infection as do some strains that are more pathogenic (32). Fluorescent *H. pylori* bacteria were readily visible in these glands after the isolation process, with clear spiral morphology (Fig. 1A and B). We observed a range of bacterial numbers in these glands, ranging from no bacteria to low or high numbers of bacteria, achieving as high as 200 bacteria/gland, as detailed in the next section (Fig. 1A). The bacteria were generally dispersed throughout the gland (Fig. 1A and B; see Movie S1 in the supplemental material). Furthermore, we determined that fluorescent protein expression was stable throughout the infection period for all our time points up to 6 months by analyzing the fluorescent signal of bacterial colonies in mouse output plates (see Fig. S1B in the supplemental material). We also verified the same colonization parameters for GFP-expressing strains compared to parental strains shown at 2 weeks postinfection (see Fig. S1C in the supplemental material). These findings demonstrated that the BLIG approach can be used to analyze the kinetics and properties of bacterial gland colonization.

To accomplish a detailed quantification of the *H. pylori* population, we determined how many glands are present in the mouse stomach corpus and antrum. Tissue in each of these regions is comprised of distinct epithelial cell types and appears to present unique challenges to *H. pylori* (27, 33, 34). Previous work had reported that the density of antral glands averaged 390 glands per mm^2^ (35). We thus combined this number with our own measurements of the tissue size to calculate that the antrum has 9,360 ± 780 glands and that the corpus has 20,410 ± 1,369 glands, assuming the same gland density in both regions (Fig. 1C). To provide a second measure of these numbers and to determine whether the gland densities in both regions were similar, we employed a saturating gland isolation procedure in which we repeatedly isolated glands from corpus or antral tissues (Fig. 1C). This second approach revealed that the antrum had 8,139 ± 361 glands, the corpus had 19,065 ± 2,031 glands, and the total stomach had 27,204 ± 2,228 glands. The two approaches yielded similar numbers, suggesting both that our estimates were reasonable and that each region had a similar gland density. For our subsequent analyses, we used the numbers obtained from the saturation gland isolation method to calculate the number of bacteria inside gastric glands.

### *H. pylori* colonizes both inside and outside glands efficiently within the first month of infection but to different extents in the corpus and antrum.

**FIG 2:**
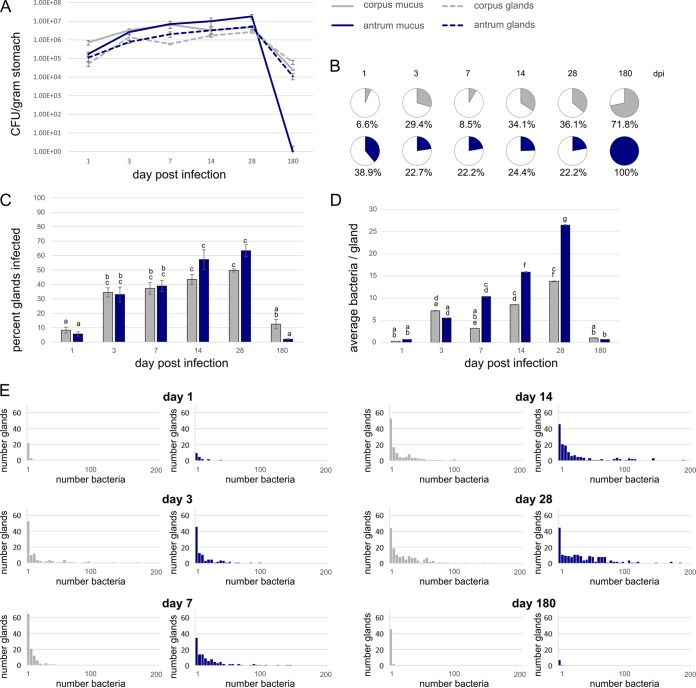
Gland colonization occurs in both the corpus and antrum and proceeds with distinct kinetics. Mice were orally infected with 10^8^ GFP-expressing *H. pylori* SS1. At the indicated times, stomachs were collected and analyzed for extragland (“mucus”) and intragland bacterial numbers. For all panels, the corpus numbers are indicated in gray and the antrum numbers are indicated in blue. (A) CFU/gram outside the glands (mucus) (solid line) and inside glands (dashed line) at the indicated time points after infection; (B) pie charts showing the percentage of the population within the glands (colored) at each time point (dpi, days postinfection); (C) percentage of glands infected over time; (D) average number of bacteria found inside the glands from 100 glands each at the indicated time points; (E) distribution of bacteria in the infected glands in the corpus (gray) and antrum (blue) over time. In panel E, data are binned by 5, e.g., the first bar shows glands with 1 to 5 bacteria, the second shows glands with 6 to 11 bacteria, etc. Glands with zero bacteria were excluded from this analysis. Statistical differences are indicated by different lowercase letters above the bars (*P* < 0.05) as analyzed by one-way analysis of variance (ANOVA) and the Tukey test.

Our first goal was to compare the kinetics of intra- and extragland colonization between the corpus and antrum (Fig. 2A). Mice were orally infected with *H. pylori* expressing GFP, and the numbers of *H. pylori* were examined over time points of 1 to 180 days after infection. In the first day of infection, the corpus was colonized on a per gram basis to slightly higher levels than the antrum (Fig. 2A), consistent with previous reports (34). Few bacteria were within the corpus glands, in contrast, even though it was colonized initially with high numbers of bacteria (Fig. 2A and B, 1 day). Less than 10% of the corpus population was in the glands, while ~30% of the antral population was within glands at these early times (Fig. 2A and B). These findings suggest that early infections in the corpus and in the antrum are substantially extragland dominant, while those in the antrum display somewhat more gland occupation.

During the next phase of infection, from 7 to 28 days postinoculation, bacterial numbers increased in both the corpus and antrum, inside and outside the glands. In the antrum, the extra- and intragland colonization loads increased substantially to levels that exceeded the corresponding corpus loads by 7 days postinfection. The antrum population remained higher than the corpus population for the first month (Fig. 2A). The number of *H. pylori* inside the glands generally increased in both corpus and antrum in parallel to the overall population increase, although the antral gland population had a somewhat more substantial increase (Fig. 2A). The percentage of the population that was within the glands stayed fairly steady throughout this period (Fig. 2B). This observation suggests that both corpus and antrum glands are permissive for *H. pylori* growth and colonization, although the antral glands appear to be more conducive to rapid multiplication.

After 6 months of infection, total and gland colonization levels decreased by 100- to 1,000-fold. The total colonization numbers arrived at levels that were below that of a 1-day infection. The extragland *H. pylori* population was eliminated in the antrum and nearly eliminated in the corpus, leading to a population that was almost exclusively localized in glands (Fig. 2A and B).

We confirmed these findings using a second set of infections with 100-fold-lower doses (see Fig. S2 in the supplemental material). Consistent with the high-dose infections, we observed a rapid extra- and intragland colonization within the first week of infection, with maximum numbers reached after 2 weeks. As seen with the high dose, antral colonization became dominant compared to corpus colonization and a somewhat higher percentage of antral glands was occupied using this regime. This infection was carried out to 60 days postinfection, and there was no reduction in bacterial numbers (Fig. S2A). These data therefore suggest that the trigger that reduces bacterial colonization acts at a time point between 2 and 6 months.

### *H. pylori* spreads rapidly to colonize glands during the acute phase of infection.

To gain insights into the *H. pylori* intragland population dynamics, we determined the percentage of infected glands and the number of bacteria per gland. At the first time point, only a few glands were occupied by *H. pylori* in both the corpus and the antrum, at least in part due to the fact that only a small percentage of the infecting dose had colonized the stomach (Fig. 2C). The vast majority of the inoculum had been eliminated, apparently with the regular stomach emptying (see Fig. S3 in the supplemental material). The number of corpus glands colonized (8%) was slightly but not significantly higher than the number of antral glands colonized (6%) (Fig. 2C). Between days 1 to 3 postinfection, there was a significant rise in the percentage of glands infected from ca. <10% to ca. 35%. The percentage of glands infected increased in both the corpus and antrum, and at each time point up to 1 month, these values were not significantly different from each other. After day 3, the infection of new glands slowed. The 28-day time point showed the highest number of glands infected with ~50% in the corpus and ~60% in the antrum (Fig. 2C). These data suggest that substantial new gland colonization takes place within the first week after infection, with the first few days playing a critical role in both regions. Similar results were seen with the low-dose infections (Fig. S2). At 6 months, the percentage of infected glands dropped dramatically, mirroring the overall bacterial population drop. At this time, significantly more corpus glands (12.5%) were infected over antral glands (2%) (Fig. 2C).

We also analyzed how the number of bacteria in each gland changed over time. The intragland bacterial number increased steadily and significantly over the first month of infection (Fig. 2D). The corpus gland population reached a maximum of 186 bacteria inside an individual gland and an average of 28 bacteria/gland after 28 days of infection (Fig. 2D). In contrast, the antral glands achieved significantly higher numbers at the same time, with a maximum of 209 bacteria inside an individual gland and an average of 26 bacteria/gland (Fig. 2D) after 28 days of infection. Most of the glands, however, had only one to five bacteria in them in both regions (Fig. 2E). At the latest time point, 180 days after infection, bacterial numbers inside individual glands dropped significantly in both regions, to below five bacteria/gland (Fig. 2D). The latter outcome suggests that both types of glands allow early high-level *H. pylori* numbers and that both also become restricted during chronic infections.

### *H. pylori* colonization protects from secondary gland infections.

**FIG 3:**
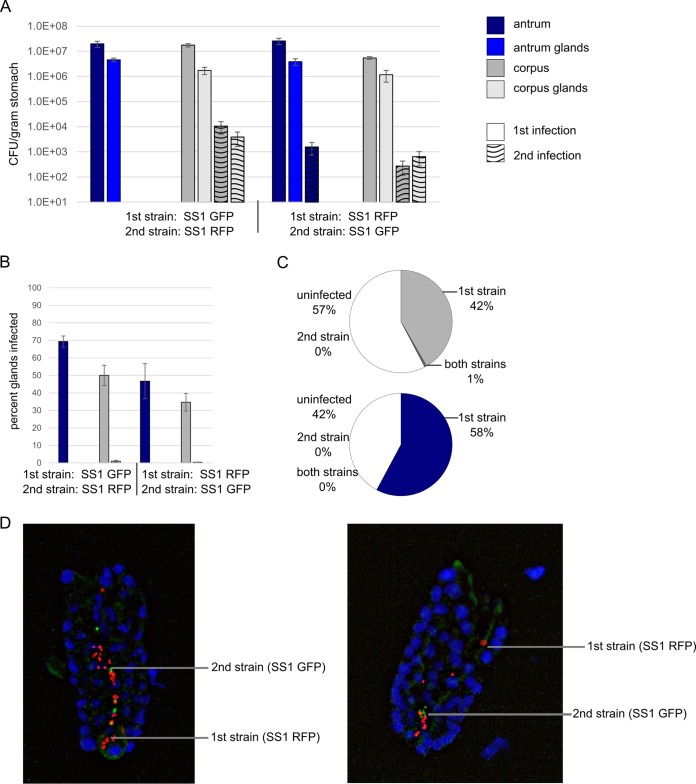
Corpus glands are permissive for subsequent gland colonization while antral glands are not despite both regions sustaining a significant fraction of unoccupied glands. Mice were orally infected on day 1 with the first strain and on day 14 with the second strain. Total and gland colonization was assessed on day 28. Strains were distinguished based on GFP or RFP expression. One experiment used SS1 GFP as the first strain and SS1 RFP as the second strain (left panel), and the other experiment used SS1 RFP as the first strain, and SS1 GFP as the second strain (right panel). Corpus numbers are shown in gray, and antrum numbers are shown in blue. (A) *H. pylori* colonization levels in total tissue (darker bars) and inside glands (lighter bars) as indicated in the key. Numbers for the first infection and second infection are shown as indicated in the key. (B) Percentage of glands colonized with the first or second strain after 28 days. Corpus numbers are shown in gray, and the antrum numbers are shown in blue. The first strain is shown in clear filled bars, and the second strain is shown in stippled bars. (C) Distribution of glands that are uninfected or glands that contain only the first strain (strain 1), only the second strain (strain 2), or both. (D) Images of glands colonized with both the red and green bacteria show that the two strains are interspersed.

We were curious whether the observed percentage of glands infected was the maximum reachable (Fig. 2C) or whether an additional *H. pylori* dose might allow empty glands to be colonized. We thus used a sequential infection model, where a high-dose inoculum was given on day 1 and a secondary high-dose inoculum was given on day 14. These time points were selected based on the saturation of the colonization observed (Fig. 2A). To be able to distinguish the two infecting strains, we infected the mice with combinations of isogenic GFP- and RFP-expressing strains. We found that the secondary infecting strain was substantially impaired for colonization (Fig. 3A), achieving numbers that were more than 1,000 times lower than the primary strain at day 28, despite being given at the same high doses (Fig. 3A). The secondary infecting strain had more substantial defects in colonizing the antrum compared with the corpus (Fig. 3A). In the antrum, the glands had no secondary strain bacteria present, and the tissue overall had very little (Fig. 3A). In terms of gland occupancy, the secondary strain was found in less than 1% of the corpus glands and none of the antrum glands (Fig. 3B). When we examined whether the secondary infection was found in glands that contained the primary infection or on its own, we discovered that it was always together with the primary strain (Fig. 3C and D). This finding suggested that the secondary strain did not colonize empty glands. In the few cocolonized corpus glands, they were all colonized to medium-high levels of >10 bacteria/gland. These findings suggest that empty glands have properties that render them difficult to colonize and that a primary infection can substantially inhibit subsequent infections by colonizing available glands to near saturation.

### Bacterial chemotaxis increases the number of glands infected but is largely dispensable for multiplication within glands.

Previous work had shown that the bacteria in glands were affected by the ability to utilize specific nutrients and bacterial chemotaxis (5, 7, 11). It was not clear, however, whether chemotaxis promoted the ability to travel to the glands or the ability to grow within the glands. We therefore employed our isolated gland methodology to gain quantitative information about the role of chemotaxis in the gland colonization process.

**FIG 4:**
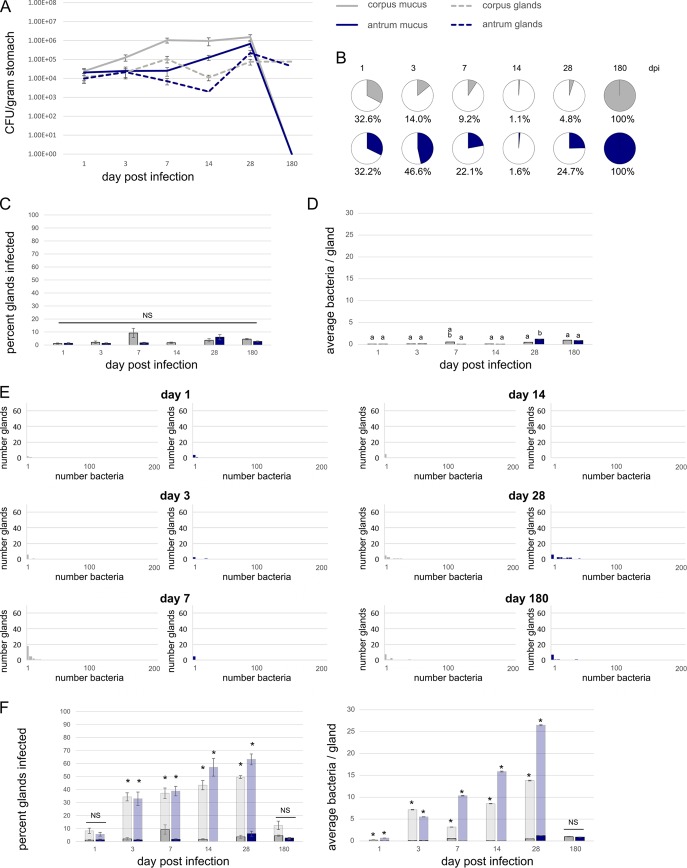
Chemotaxis plays a critical role in gland colonization. Mice were infected with GFP-expressing Che^−^ *H. pylori* SS1. At the indicated times, stomachs were collected and analyzed for total bacterial numbers and gland bacterial numbers. For all panels, corpus numbers are shown in gray, and antrum numbers are shown in blue. (A) CFU/gram stomach outside the glands (mucus) (continuous line) and inside glands (dashed line) at the indicated time points after infection. (B) Pie charts show the percentage of the population within glands (colored) and outside the glands at each time point. (C) Percentage of glands infected over time in the corpus and antrum. (D) Average number of bacteria found inside infected glands from 100 glands each at the indicated time points. (E) Distribution of bacteria in the infected glands over time. Data in panel E are binned by 5, e.g., the first bar shows glands with 1 to 5 bacteria, the second shows glands with 6 to 11 bacteria, etc. Glands with zero bacteria were excluded from this analysis. (F) Comparison of wild-type and chemotaxis mutant in the percentage of glands infected and average number of bacteria inside glands (replotted from Fig. 2C and 4C and Fig. 2D and 4D). Statistical differences are indicated by different lowercase letters above the bars (*P* < 0.05) as analyzed by one-way ANOVA and the Tukey test in panels C and D and by an asterisk as analyzed by one-way ANOVA (*P* < 0.05) in panel F. Values that are not statistically different (NS) are indicated.

We constructed isogenic *H. pylori* GFP- or RFP-positive strains that are nonchemotactic (Che^−^) and used these strains to infect mice as described for the wild-type (WT) strain above. Fluorescent protein expression was maintained in the Che^−^ strain, as with the WT, and this strain had similar chemotaxis and colonization properties as its parent (see Fig. S1 in the supplemental material). Initially, 10- to 100-fold-less Che^−^ *H. pylori* than WT *H. pylori* colonized the stomach at day 1 postinfection in both the extra- and intragland regions (compare Fig. 4A with Fig. 2A). Calculations suggest that very large numbers of the Che^−^ strain were eliminated from the stomach shortly after the infection, giving rise to few bacteria that seed the gastric tissue (Fig. S3). The total bacterial numbers of the Che^−^ strain increased 10-fold between day 1 and day 28 (Fig. 4A), an increase that was comparable to that of WT (Fig. 2A). The colonization kinetics of the Che^−^ strain were slowed, however, particularly in the glands. This phenotype was strongly evident at the 14-day time point, when less than 5% of the population was within glands (Fig. 4A and B). The Che^−^ strains additionally were unable to multiply efficiently in the antrum as the WT did. Che^−^ strains, in contrast, stayed dominant in the corpus throughout the infection period (Fig. 4A). Similar to the results with WT, we observed a reduction in the numbers of the Che^−^ strain at the 6-month time point, when the population collapsed to an entirely intragland state. This reduction resulted in the Che^−^ infection having colonization numbers that were similar to those of the WT infection (Fig. 4A and 2A).

We also analyzed the gland colonization patterns of the Che^−^ strain. Without chemotaxis, the fraction of glands infected was significantly below that of the WT, hovering below 10%. Unlike the WT, this percentage stayed mostly constant over the entire infection period of 180 days (Fig. 4C and F). This finding suggests that chemotaxis is required for efficient targeting of new glands, although some glands can be reached in a chemotaxis-independent manner. In confirmation of this idea, low-dose Che^−^ infections had great difficulty occupying the glands (see Fig. S4 in the supplemental material), suggesting that Che^−^ bacteria may instead rely on more stochastic processes that are promoted by large populations. Tracking the bacterial numbers inside glands, we observed that the numbers of Che^−^ bacteria increase only at later time points, 28 and 180 days postinfection, and remain significantly lower than the number of WT bacteria throughout infection at points up to 28 days (Fig. 4D and F). The maximum bacterial numbers achieved by a Che^−^ mutant inside the glands was about four times lower than that of WT at 28 days postinfection but was not significantly different from that of WT at 180 days postinfection (Fig. 2D and 4D).

### Nonchemotactic *H. pylori* bacteria cannot prevent a secondary infection.

As shown above, an established WT primary infection largely prevents colonization by a second infection (Fig. 3). In that case, the percentage of glands colonized had reached the equilibrium level. Given that Che^−^ infections never achieve that level of gland coverage, we were interested in whether a primary infection with a Che^−^ mutant would similarly prevent a secondary infection or whether a secondary infection with the WT strain might be able to completely abolish the less-fit primary infection in a particular niche(s). To examine these questions, we performed sequential infection experiments with GFP- or RFP-expressing Che^−^ *H. pylori* as the primary infection, and RFP-expressing and GFP-expressing WT *H. pylori* as the secondary infection.

**FIG 5:**
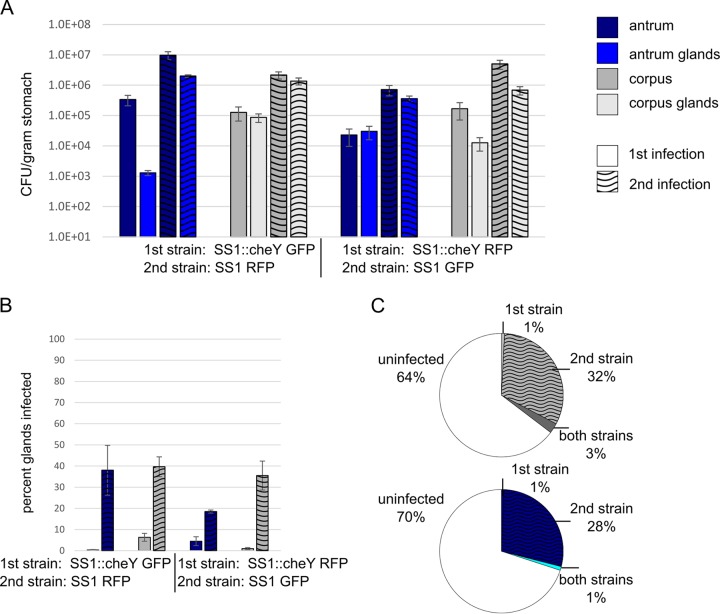
Established colonization by Che^−^ strains does not prevent a secondary wild-type infection. Mice were infected and analyzed as d escribed in the legend to Fig. 3, except the primary infection was with an isogenic Che^−^ strain. For all panels, corpus numbers are shown in gray, and antrum numbers are shown in blue. (A) Bacterial colonization levels in total tissue and inside glands; (B) percentage of glands colonized with the first strain or the second strain after 28 days; (C) distribution of glands that are uninfected, contain only the Che^−^ first strain, contain only the WT second strain, or contain both.

Our first observation was that the primary Che^−^ bacterial infection was not completely displaced by a secondary WT bacterial infection (Fig. 5A). The Che^−^ mutant retained colonization in both the corpus and antrum (Fig. 5A). The number of the primary Che^−^ strain was about 5 times below that of a single Che^−^ bacterial infection in both the corpus and antrum, suggesting that some of the Che^−^ strain had been cleared by the WT bacterial infection (compare Fig. 4A to Fig. 5A). In contrast to the WT-WT bacterial infections, the secondary infection was able to establish a robust colonization. This colonization led to substantially higher numbers of WT bacteria than of the Che^−^ bacteria from the primary infection in both the total stomach and in the glands (Fig. 5A). These data therefore suggest that the Che^−^ mutant is not able to prevent a secondary infection, likely because the majority of glands remain empty. Consistent with this idea, we observed an increase in the total number of glands infected when a WT bacterial infection followed a Che^−^ bacterial infection compared to Che^−^ bacterial infection alone (Fig. 5B). At day 28, the Che^−^ strain was found in less than 10% of the glands in the corpus and antrum, while the secondary WT bacterial infection colonized 20 to 40% of antrum glands and 30 to 40% of corpus glands. These numbers were slightly lower than observed for a WT-only bacterial infection after 14 days (Fig. 2B). Therefore, precolonization with a Che^−^ strain seems to affect the future colonization outcome but certainly did not prevent WT colonization. When examined for whether the secondary WT bacterial infection colonized empty glands or glands colonized with Che^−^ strain, we found that the majority of the secondary WT bacterial infection was located in glands that contained only WT bacteria in both the corpus and the antrum (Fig. 5C). We did observe, however, that the secondary infection cocolonized with the primary Che^−^ infection in both regions, with somewhat more double colonized glands in the corpus (Fig. 5C). As observed with WT-WT bacterial infections (Fig. 3), a substantial fraction of the glands remained uncolonized. These data combined with the WT-WT bacterial data (Fig. 3C) indicate that a gland containing bacteria does not exclude entrance of other *H. pylori*. The data additionally show that bacterial gland populations are not necessarily clonal and instead can result from a combination of new bacterial entrance and division. Last, our data support the idea that a stomach that is not colonized to saturation by fully fit *H. pylori* can still be colonized by a secondary infection. Therefore, the presence of *H. pylori* itself does not prevent future infections but instead requires fit *H. pylori* that fully occupy the glands.

## DISCUSSION

Employing a powerful new gland analytical method we developed here termed BLIG, we report the kinetics of bacterial biogeography in the mammalian host. Using *H. pylori* as our model, we found that bacteria initiate host colonization inside and outside the glands, with very early gland colonization appearing mostly stochastic. The microbial population expands both inside and outside the glands in a chemotaxis-dependent manner. *H. pylori* multiplies more rapidly in some tissue—the antrum—compared to the corpus, suggesting that these regions present distinct challenges. Bacterial levels reach a maximum after 2 to 4 weeks of infection, with the antrum more heavily colonized than the corpus. At the height of infection, bacterial populations reach more than 200 bacteria/gland, and antral glands support a greater quantity of *H. pylori* than corpus glands do. Infections never occupy 100% of the glands and instead leave ~50% unoccupied. Similar observations about the percentage of glands occupied have been reported using microscopy (11), confirming the accuracy of our BLIG approach. Sequential infections showed that these unoccupied glands are unable to be colonized, suggesting that they differ from the colonized glands in some as-yet unknown way. Furthermore, we determined that the gland populations are not exclusively clonal and can arise from new bacteria joining a colonized gland.

During the chronic phase of infection, *H. pylori* populations undergo a substantial collapse to a setting with both few glands infected and few bacteria/gland. Interestingly, this long-term gland population was achieved whether the bacteria were chemotactic or not, as long as a sufficient dose was given. Last, we report that existing gland-based infections can protect against subsequent ones, providing insights into bacterial properties that confer self-colonization resistance.

### BLIG gland analysis represents a powerful technique that can be applied to other gastrointestinal tract bacteria.

Our method represents a robust way to quantify bacterial colonization and biogeography within the gastrointestinal tract. This method should be readily transferable to bacteria that colonize other portions of the gastrointestinal tract, including the intestine, as intestinal crypt isolation is well established for the preparation of intestinal organoids (30). Analyses such as these may have broad implications for future personalized microbiota therapeutics, the goal of which is to replace harmful microbiota members with beneficial ones. Such an approach would benefit from a strong understanding of the host and bacterial physiological features that influence gland colonization, such as would be gained from the BLIG approach. Ultimately, understanding gland colonization will allow manipulations that increase the number of beneficial bacteria while decreasing the number of pathogenic bacteria inside the gastrointestinal tract.

### Gland colonization proceeds rapidly to saturation.

*H. pylori* initially occupies only a few glands, with comparable numbers between WT and Che^−^ infections (compare Fig. 2 with Fig. 4). These data thus suggest that gland entrance in the first 24 h might be based on random processes rather than active ones such as chemotaxis. Not all gastrointestinal gland-colonizing bacteria are motile or chemotactic, consistent with the idea that motility aids but is not required for this process (5, 10). From days 1 to 14, bacterial numbers rapidly increase both inside and outside the glands, in both the corpus and antrum. It is not yet clear whether there is movement between these populations; however, gaining access to glands is greatly assisted by chemotaxis, suggesting that there may be active movement from the extra- to intragland niches.

By 14 to 28 days postinfection, the antrum is more highly colonized than the corpus in both the percentage of glands colonized and in higher numbers of bacteria/gland. *H. pylori* had been shown previously to undergo ~2-fold higher replication rate in the antrum than in the corpus (34), suggesting that this niche is quite favorable during the acute phase. The time period from 14 to 28 days postinfection is commonly used in *H. pylori* mouse work, and analysis in this time window would conclude that mouse infections are antral dominant.

### A single-dose wild-type bacterial infection can saturate available glands to afford self-colonization resistance but does not occupy all the glands.

A single-dose infection is sufficient to saturate the bacterial numbers as well as the percentage of glands infected. Many glands were uncolonized even at this saturation stage, suggesting that glands differ from each other in as-yet unknown ways. We speculate that these glands have differences that prevent their colonization, such as physical differences that block *H. pylori* from entering them, or that they have immune cells nearby. The nature of such differences, however, is not yet known.

A primary *H. pylori* infection almost completely prevented a secondary infection. This phenomenon is a form of colonization resistance that has been observed previously in *H. pylori* as well as for *Bacteroides* species (5, 27), in which the same bacterium prevents subsequent infections by members of its own genus. In both *Bacteroides* and *H. pylori*, self-colonization resistance relies on prior colonization by optimally fit bacteria that can fully occupy their niches (5). Non-self-colonization resistance, in contrast, occurs when particular microbiome members prevent infection by other microbial species (36, 37). Non-self-colonization resistance has been shown to occur by several mechanisms, including induction of the interleukin 22 (IL-22) immune axis or secondary bile metabolites (38, 39). Whether these mechanisms operate during self-colonization resistance or in the stomach, however, is not known.

Colonization resistance has important implications for medical treatments of *H. pylori* and other chronic bacterial infections. People who are infected with a particular strain first—one that is fit but nonpathogenic—might have a natural protection from future *H. pylori* infections with potential harmful strains. While *H. pylori* can lead to diseases such as gastritis, gastric ulcers, and gastric cancer (17, 40–42), the majority of people harboring *H. pylori* have been found to be asymptomatic. In some cases, these infections are even advantageous in preventing respiratory diseases such as asthma or autoimmune diseases (16, 43, 44). Therefore, colonization with *H. pylori* strains that are fit but lack key virulence factors might prevent subsequent infections with strains that would promote gastric diseases.

### Chronic infection is characterized by small gland-localized bacterial populations.

During the chronic infection phase, *H. pylori* populations are localized in glands and characterized by low numbers, dropping below those seen in the initial infection time points (Fig. 2). These observations suggest that the stomach physiology, immune response, and/or bacterial physiology changes over the course of the infection to severely restricted *H. pylori* numbers, particularly in the antrum. Multiple changes are known to happen in the 2- to 6-month infection window, including development of an immune response and recruitment of immune cells, but we do not yet know how these precisely affect *H. pylori* (16, 45, 46). Interestingly, both WT and Che^−^ bacteria end up at similar final settings: entirely gland localized in the antrum and nearly so in the corpus, an average of 2 to 8 bacteria/gland, and 5 to 15% of glands infected. WT bacterial populations undergo a more severe restriction than the Che^−^ bacterial populations do, because the WT bacterial infections start from higher bacterial loads. Furthermore, WT bacterial infections have been shown to elicit substantially more inflammation then Che^−^ bacterial infections do (28, 46, 47). Thus, one idea is that immune-related parameters restrict *H. pylori* to low numbers and few glands.

Our data suggest that the gastric glands generate the crucial niche for long-term maintenance of *H. pylori* infections. One question is how such low bacterial populations might be able to maintain robust colonization and colonize new glands. Individual gastric cells have varied life spans of days to months, leading to continuous renewal of the cells composing the gland (48). How frequently new glands arise is not as clear. Early work by Lee (35) observed glands with branched bases, which were suggested to be glands in the process of splitting. This observation led to the idea that one gland may split to give rise to a second, or daughter, gland. In this case, the *H. pylori* population may split with the glands and simply go along with the daughter gland. Another alternative, however, is that generally the glands themselves have very long half-lives, and only individual cells turn over in healthy tissue. Such an idea is supported by stem cell tracing experiments in which LgrV-positive cells were marked for a short time, and then the number of glands bearing these marked cells was observed over time (49). There was not an appreciable change between 4 and 620 days, suggesting that the glands themselves are relatively stable. Thus, one possibility is that glands are retained on a long time scale, and therefore that *H. pylori* needs only to multiply minimally. Future experiments to measure the *H. pylori* growth rate will be informative for this aspect of bacterial biology.

### Chemotaxis aids initial seeding and rapid acute-phase gland expansion but is not required for chronic colonization.

Chemotaxis is an important colonization factor for many gastrointestinal-tract-colonizing bacteria in addition to *H. pylori*, including the human pathogens *V. cholerae*, *Salmonella enterica* serovar Typhimurium, and *C. jejuni* (23, 24, 50–52). We report here that chemotaxis greatly aids the initial bacterial colonization step, in agreement with previous findings (27), allowing an additional 0.02% of the input dose to colonize; with a dose of 10^7^, this represents an additional 2,000 bacteria that make it to the mucous layer.

Chemotaxis also plays critical roles in the early stages of bacterial multiplication, with Che^−^ mutants showing strong deficiencies in the antrum both inside and outside the glands. Interestingly, the relative percentage of the bacterial population inside the glands was the same for both WT and Che^−^ strains (Fig. 2 and 4), but the Che^−^ strains had overall lower numbers and were never able to fully occupy the glands. This inability was further highlighted by sequential infections, where Che^−^ bacteria left a significant proportion of glands available. Late in infection, Che^−^ and WT bacterial infections did not differ substantially in terms of bacterial numbers in any compartment. This outcome was somewhat surprising and raises the question of why wild-type *H. pylori* multiply to such high numbers if that response is not needed for chronic infection. However, our data support that chemotaxis plays only a minor role during the chronic stage.

In summary, we report that *H. pylori* populations ascend to high levels both outside and inside the gastric glands during the acute infection phase. Achieving these high numbers depends on chemotaxis. However, even at the maximal infection state, only about half of the gastric glands are colonized by *H. pylori*, suggesting there are as-yet unappreciated parameters that prevent gastric gland colonization and that possibly can be harnessed in the future to control bacterial infections. During the chronic state, *H. pylori* numbers collapse down to a modest ~10% of glands infected with only a few bacteria/gland. Achieving this state is chemotaxis independent. Whether other chronically colonizing bacteria display similar population dynamics remains to be elucidated, but the BLIG approach developed will allow the detailed study of the gland and crypt populations in other portions of the gastrointestinal tract. Eventually, understanding the parameters that promote chronic colonization will allow the successful design of microbial therapeutics that maintain long-term mammalian colonization.

## MATERIALS AND METHODS

### *H. pylori* strains and growth conditions.

*H. pylori* strain SS1 (53) and its isogenic SS1 Che^−^ (Δ*cheY*::*cat-102*) mutant (27) were used for all studies. These strains were grown at 37°C, 5% O_2_, and 10% CO_2_ on solid media consisting of Columbia blood agar (BD Diagnostics, Fisher Scientific) with 5% difibrinated horse blood (Hemostat Laboratories, Dixon, CA), 50 µg/ml cycloheximide (VWR), 10 µg/ml vancomycin, 5 µg/ml cefsulodin, 2.5 U/ml polymyxin B (all from Gold Biotechnology, St. Louis, MO), and 0.2% (wt/vol) β-cyclodextrin (Spectrum Labs, Gardena, CA) (CHBA) or in liquid medium consisting of brucella broth (BBL) medium supplemented with 10% fetal bovine serum (FBS) (Life Technologies) (BB10). To generate fluorescent *H. pylori*, strains were transformed with either pTM115 (*ureAp*-GFP *aphA3* Km^r^) (54, 55) or pTM115-RFP (*ureAp*-RFP *aphA3* Km^r^). Both plasmids express the fluorescent proteins from a 750-bp fragment that includes the urease promoter and spans from bases 77956 to 78706 using *H. pylori* 26695 numbering (56). pTM115 is similar to pTM117 (57) with the addition of the urease promoter. pTM115-RFP was generated by removing the *gfp* gene of pTM115 by cutting with PstI, rendering the plasmid blunt ended, and then digesting with SalI. The *rfp* gene was subcloned from pDS-Red (Clontech) as a SpeI (blunted)-SalI fragment, which was then ligated to the cut pTM115. For *H. pylori* transformation, plasmids were methylated with *H. pylori* SS1 cell extract as described previously (58), and then used in standard natural transformation protocols (59, 60) with 15 µg/ml kanamycin for selection in CHBA.

### Ethics statement.

The University of California Santa Cruz Institutional Animal Care and Use Committee approved all animal protocols and experiments (protocol OTTEK1505). All animal procedures used were in strict accordance with the *Guide for the Care and Use of Laboratory Animals* (61). Female C57BL/6N mice (*Helicobacter* free; Charles River) were housed at the University of California Santa Cruz animal facility.

### Animal infections and *H. pylori* colonization load calculations.

Mice were 6 to 8 weeks old at the time of *H. pylori* infection. Animals were orally infected via a 20-gauge 1.5-in. feeding needle (Popper) with 500 µl containing *H. pylori* grown to mid-exponential phase (optical density at 600 nm [OD_600_] of ~0.3) in BB10 medium. After the infection period, the animals were sacrificed via CO_2_ narcosis, and the stomach was removed by cutting at the stomach-esophageal junction and the antrum-duodenum sphincter. The fore stomach was removed, and the stomach opened along the lesser curvature. The stomach was gently rinsed in 25 ml ice-cold phosphate-buffered saline (PBS) to remove food. Blood vessels and the muscle layer were removed from the nonluminal side of the tissue using microscissors (Kelly Scientific) and tweezers. The stomach was then dissected as shown in Fig. 1, separating the corpus from the antrum using the difference in tissue coloration as a marker for the border between these regions. Each piece was then cut into two, one for total bacterial number and one for gland isolation. For total bacterial number, the tissue was weighed, homogenized using the Bullet Blender (Next Advance) with 1.0-mm zirconium silicate beads, and then plated to determine the number of CFU per gram of stomach tissue on CHBA with the addition of 20 µg/ml bacitracin, 10 µg/ml nalidixic acid, and 15 µg/ml kanamycin. To enumerate bacteria expressing red or green, plates were analyzed using a Bio-Rad Chemi-Doc imaging system using settings for fluorescent antibodies at 488 and 594 nm.

Gland colonization levels were calculated by taking the average number of bacteria per gland in a sample of 100 glands, and multiplying that by the number of glands in each region. To convert to per gram stomach, we used an average weight that was obtained by dividing the total gland number for corpus glands (19,065) and antral glands (8,139) by the average weight of the stomach pieces used for gland number counting (Fig. 1). To obtain the outside-gland/mucous numbers, the gland population was subtracted from the total CFU per gram from plating.

### Gland isolation.

Glands were isolated using a protocol adapted from Mahé et al. (29). Dissected gastric tissue was cut into 1-mm^2^ pieces and incubated with slight shaking in Dulbecco’s phosphate-buffered saline (DPBS) (Millipore) plus 5 mM EDTA at 4°C for 2 h. After this period, the tissue was transferred into ice-cold DPBS containing 1% sucrose and 1.5% sorbitol and shaken roughly by hand for 2 min. The remaining large tissue pieces were allowed to settle, and 2 ml of the solution containing the glands was removed. Glands were then transferred into a clean tube, washed two times with 2 ml DPBS each time, and labeled with 10 µg/ml Hoechst DNA stain (Life Technologies). Glands were kept on ice until examined.

### Stomach measurements and gland quantification.

To determine the density of glands in the corpus and antrum, we employed two methods. For each approach, we used three uninfected mice. For the first approach, mice were prepared as described above, and after the stomach was rinsed with PBS, it was pinned down without stretching. Pictures were taken of the stomach pieces and a scale bar. The sizes of the corpus and antrum were determined using ImageJ, differentiating between the corpus and antrum by tissue color. To obtain the number of glands in these regions, the average size of the tissue piece in square millimeters was multiplied by a previously determined density of 390 glands per mm^2^ inside the antral stomach region of mice (35). For our second approach, we isolated the glands as described above using the protocol of Mahé et al. (29). Instead of a single shaking using the sucrose/sorbitol solution, we repeated this step in three sequential rounds. After each shaking, the number of glands per milliliter was analyzed by counting the number of glands for three 10-µl samples under the microscope (magnification of ×50; phase contrast). After removing the supernatant containing the glands, 2 ml of fresh sorbitol/sucrose solution was added for the next shaking round. The numbers on each shaking round decreased strongly (Fig. 1). From these data points, a curve was fitted, to extrapolate the number of glands in this tissue piece by adding the glands from each shaking round. We determined that the BLIG method was able to isolate 93% of the antral glands and 87% of the corpus glands compared to the size measurements.

### Microscopy.

Ten microliters of isolated glands that had been stained with Hoechst were placed on glass slides and visualized using a Nikon Eclipse E600 microscope with fluorescence filters for 4′,6′-diamidino-2-phenylindole (DAPI), GFP, and RFP. For each time point and infection, 100 glands each were imaged for the corpus and antrum, and the number of *H. pylori* cells inside the gland was counted manually for each gland.

## SUPPLEMENTAL MATERIAL

Movie S1  Movie showing two glands with mammalian cells (red), collected from a mouse that had been infected with GFP-expressing *H. pylori* SS1 (green). The movie of a three-dimensional (3D) view of the glands was generated using Imaris software. A Z-stack through two representative glands after 14 days of infection was imaged with the Zeiss LSM 5 Pascal confocal microscope. To differentiate and visualize the cells and bacteria, mammalian cells were labeled with deep red anthraquinone 5 (DRAQ5) and GFP-expressing *H. pylori* cells were used. Download Movie S1, AVI file, 12.1 MB

Figure S1  Fluorescent protein expression does not influence WT or Che^−^ phenotypes. (A) GFP-expressing strains and parental strains display the same phenotypes in chemotaxis soft-agar assays. Plates containing brucella broth, 2.5% FBS, and 0.35% agar were inoculated with the indicated strains and incubated at 37°C under microaerobic conditions for 5 days. (B) GFP expression is stable throughout the infection process. *H. pylori* isolated from mouse stomachs was plated on CHBA supplemented with bacitracin and nalidixic acid but with no kanamycin (red plates) and then examined for GFP expression using the Chemidoc imaging system (black and white plates, with white indicating fluorescence). The two panels on the right are magnified views of the boxed region. (C) GFP-expressing strains display the same colonization phenotype as that observed for the parental strains (27). *H. pylori* strains isolated from mouse stomach were plated on CHBA supplemented with bacitracin and nalidixic acid plus kanamycin (left) or without kanamycin (right). Download Figure S1, TIF file, 7.5 MB

Figure S2  Low-input gland colonization occurs in both the corpus and antrum compared to high-input numbers. Mice were infected with GFP-expressing *H. pylori* SS1 with a dose of 10^6^ bacteria. At the indicated times, stomachs were collected and analyzed for total bacterial numbers and gland bacterial numbers. For all panels, corpus numbers are shown in gray and antrum numbers are shown in blue. (A) CFU/gram stomach outside glands/mucus (continuous line) and inside glands (dashed line) at the indicated time points after infection. (B) Pie charts show the percentage of the population within and outside the glands at each time point. (C) Percentage of glands infected over time. (D) Average number of bacteria found inside the glands from 100 glands each at the indicated time points. (E) Distribution of bacteria in the infected glands over time. Data are binned by 5, e.g., the first bar shows glands with 1 to 5 bacteria, the second bar shows glands with 6 to 11 bacteria, etc. Glands with zero bacteria were excluded from this analysis. Download Figure S2, TIF file, 16.6 MB

Figure S3  Initial bacterial release after infection. Mice were infected with ~10^8^ bacteria (input numbers shown by black bars), and the number of infecting bacteria was determined from stomach homogenates after 24 h (output numbers shown by gray bars). Download Figure S3, TIF file, 3.1 MB

Figure S4  Low-dose infections of a nonchemotactic mutant fail to persist. Mice were infected with GFP-expressing Che^−^ *H. pylori* SS1 with a dose of 10^6^ bacteria. At the indicated times, stomachs were collected and analyzed for total bacterial numbers and gland bacterial numbers. For all panels, corpus numbers are shown in gray and antrum numbers are shown in blue. (A) Numbers of CFU/gram stomach in mucus (continuous line) and inside glands (dashed line) at the indicated time points after infection. (B) Pie charts show the percentage of the population within the glands and outside the glands at each time point. (C) Percentage of glands infected over time. (D) Average number of bacteria found inside the glands from 100 glands each at the indicated time points. (E) Distribution of bacteria in the infected glands over time. Data are binned by 5, e.g., the first bar shows glands with 1 to 5 bacteria, the second shows glands with 6 to 11 bacteria, etc. Glands with zero bacteria were excluded from this analysis. Download Figure S4, TIF file, 19.3 MB
