# Supplementary figures and images for: Spatial and Temporal Shifts in Bacterial Biogeography and Gland Occupation during the Development of a Chronic Infection
Source: mBio. 2016 Oct 11;7(5):e01705-16. doi: 10.1128/mBio.01705-16 (PMC5061875; doi:10.1128/mBio.01705-16)

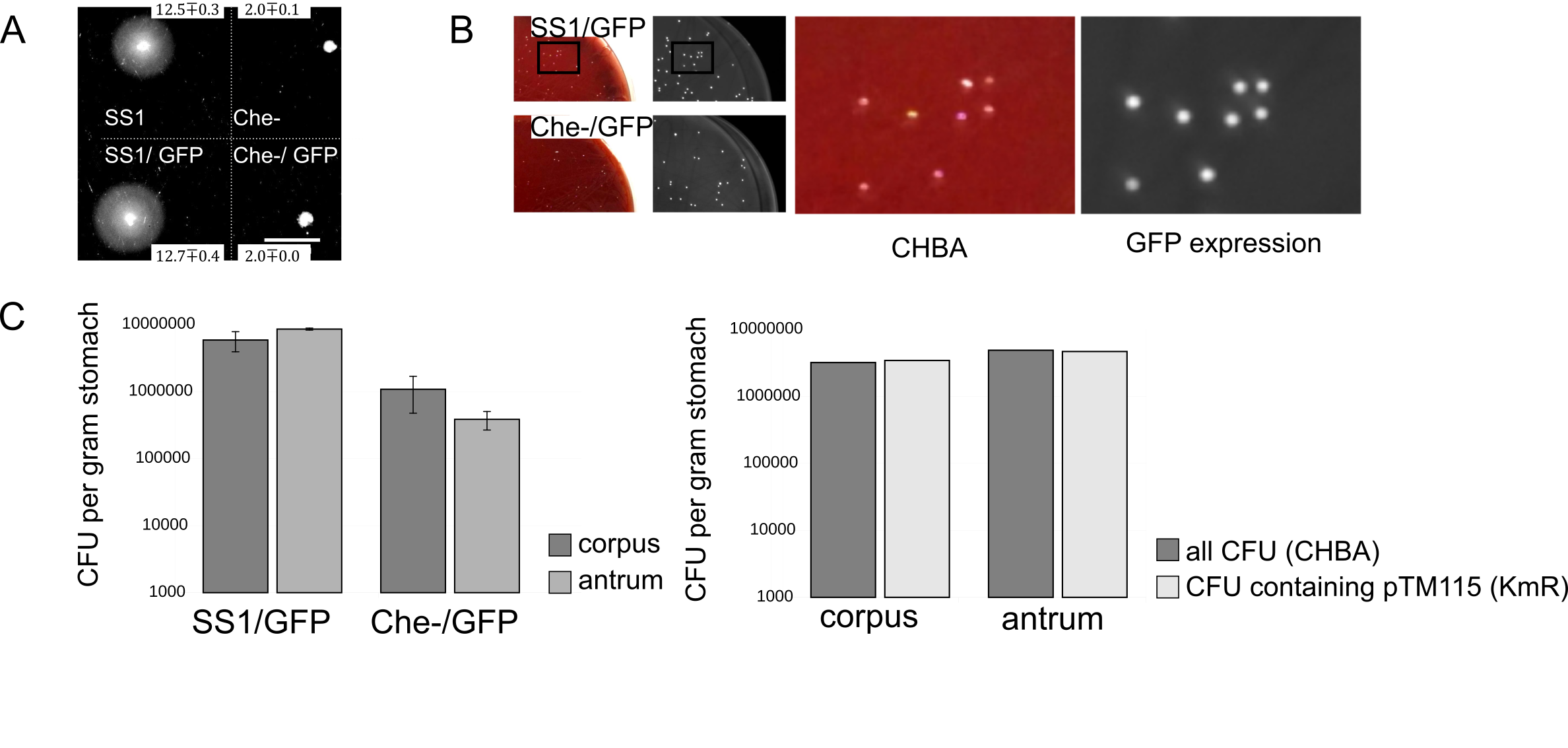

Supplement: Figure S1 — Fluorescent protein expression does not influence WT or Che− phenotypes. (A) GFP-expressing strains and parental strains display the same phenotypes in chemotaxis soft-agar assays. Plates containing brucella broth, 2.5% FBS, and 0.35% agar were inoculated with the indicated strains and incubated at 37°C under microaerobic conditions for 5 days. (B) GFP expression is stable throughout the infection process. H. pylori isolated from mouse stomachs was plated on CHBA supplemented with bacitracin and nalidixic acid but with no kanamycin (red plates) and then examined for GFP expression using the Chemidoc imaging system (black and white plates, with white indicating fluorescence). The two panels on the right are magnified views of the boxed region. (C) GFP-expressing strains display the same colonization phenotype as that observed for the parental strains (27). H. pylori strains isolated from mouse stomach were plated on CHBA supplemented with bacitracin and nalidixic acid plus kanamycin (left) or without kanamycin (right). Download [file mbo005163029sf1.tif]

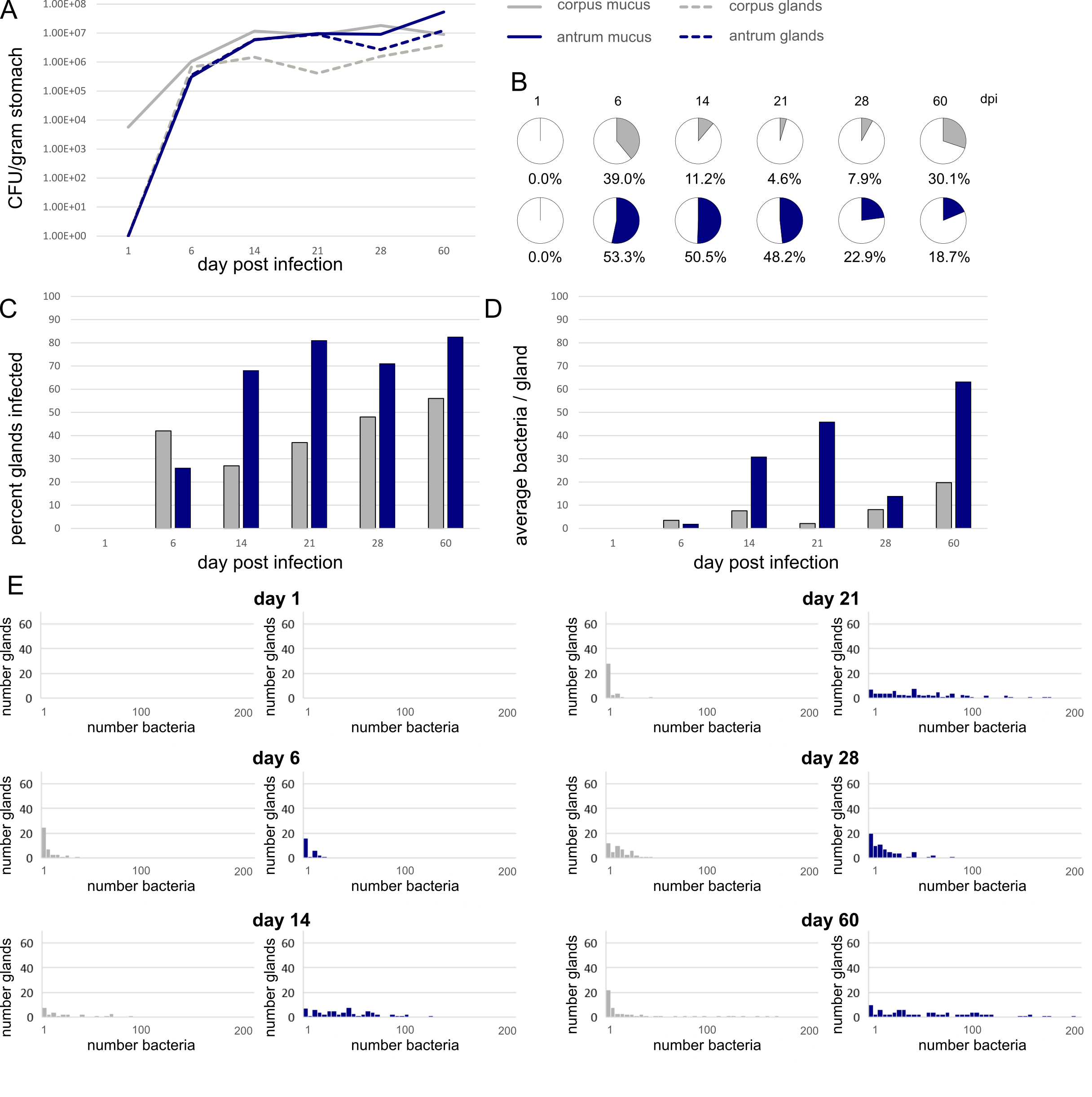

Supplement: Figure S2 — Low-input gland colonization occurs in both the corpus and antrum compared to high-input numbers. Mice were infected with GFP-expressing H. pylori SS1 with a dose of 106 bacteria. At the indicated times, stomachs were collected and analyzed for total bacterial numbers and gland bacterial numbers. For all panels, corpus numbers are shown in gray and antrum numbers are shown in blue. (A) CFU/gram stomach outside glands/mucus (continuous line) and inside glands (dashed line) at the indicated time points after infection. (B) Pie charts show the percentage of the population within and outside the glands at each time point. (C) Percentage of glands infected over time. (D) Average number of bacteria found inside the glands from 100 glands each at the indicated time points. (E) Distribution of bacteria in the infected glands over time. Data are binned by 5, e.g., the first bar shows glands with 1 to 5 bacteria, the second bar shows glands with 6 to 11 bacteria, etc. Glands with zero bacteria were excluded from this analysis. Download [file mbo005163029sf2.tif]

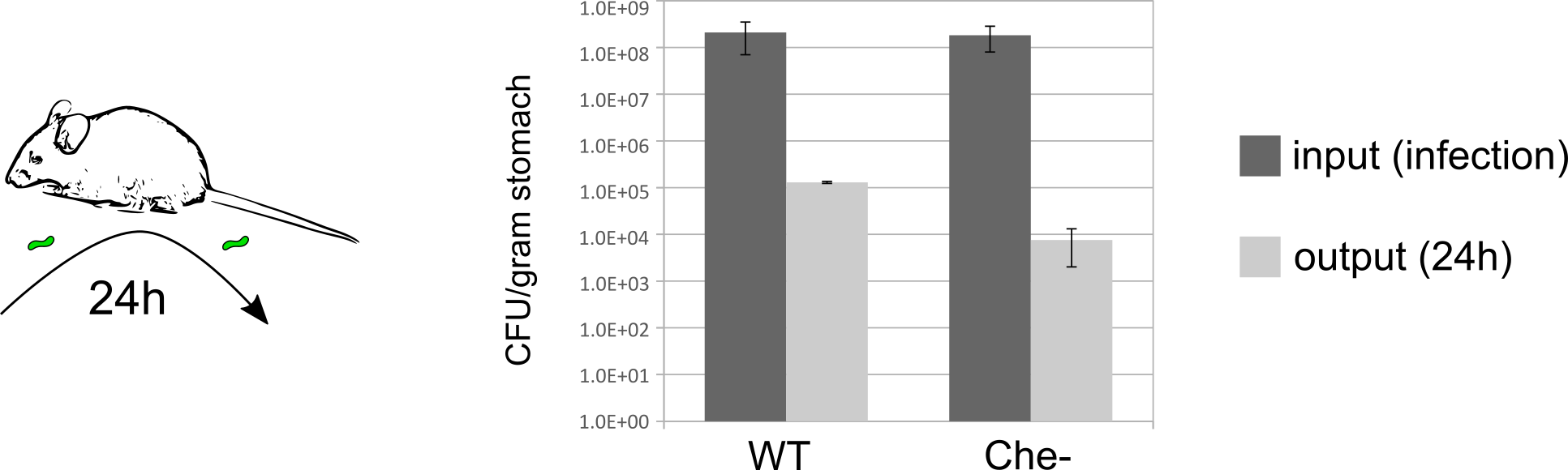

Supplement: Figure S3 — Initial bacterial release after infection. Mice were infected with ~108 bacteria (input numbers shown by black bars), and the number of infecting bacteria was determined from stomach homogenates after 24 h (output numbers shown by gray bars). Download [file mbo005163029sf3.tif]

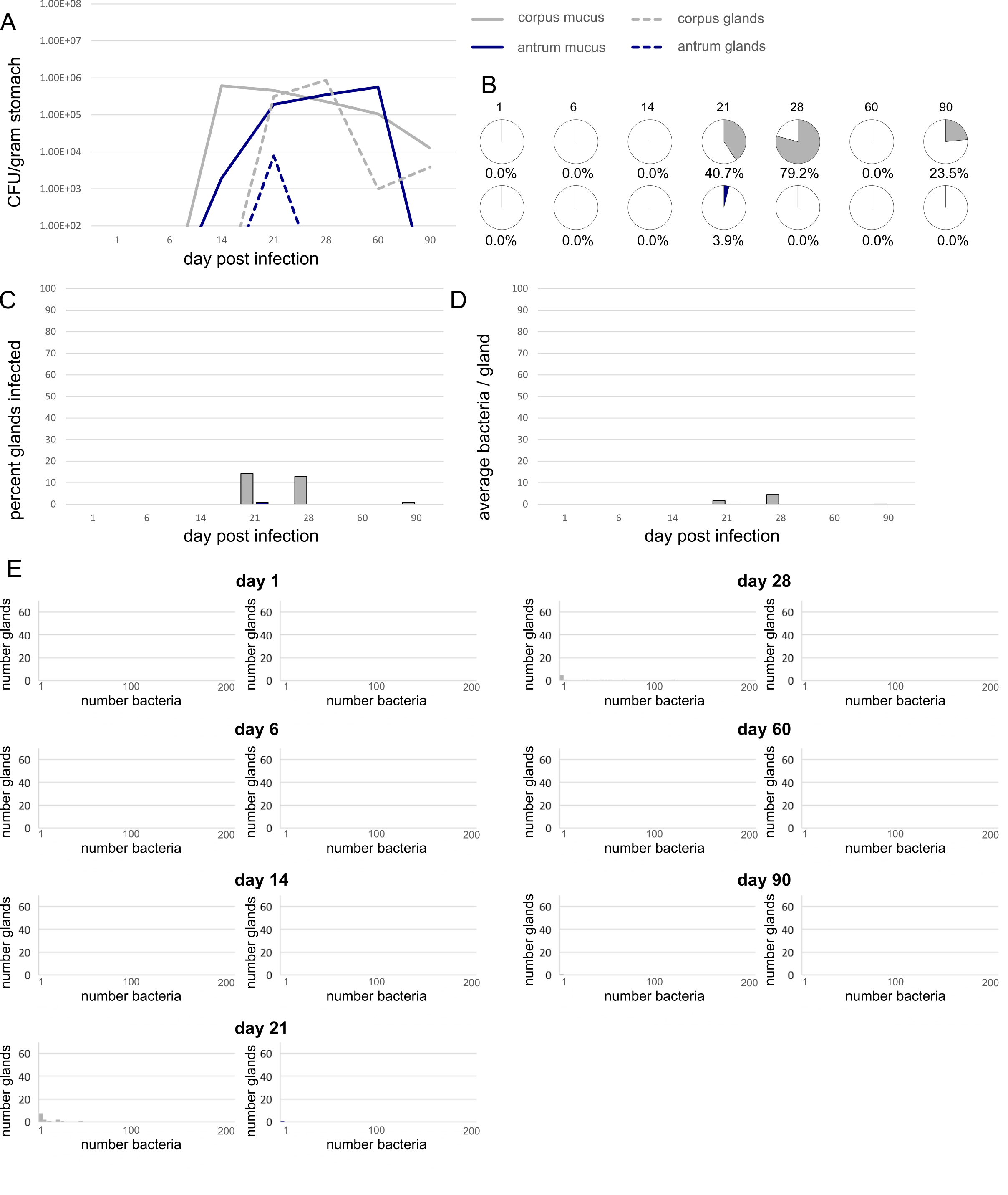

Supplement: Figure S4 — Low-dose infections of a nonchemotactic mutant fail to persist. Mice were infected with GFP-expressing Che− H. pylori SS1 with a dose of 106 bacteria. At the indicated times, stomachs were collected and analyzed for total bacterial numbers and gland bacterial numbers. For all panels, corpus numbers are shown in gray and antrum numbers are shown in blue. (A) Numbers of CFU/gram stomach in mucus (continuous line) and inside glands (dashed line) at the indicated time points after infection. (B) Pie charts show the percentage of the population within the glands and outside the glands at each time point. (C) Percentage of glands infected over time. (D) Average number of bacteria found inside the glands from 100 glands each at the indicated time points. (E) Distribution of bacteria in the infected glands over time. Data are binned by 5, e.g., the first bar shows glands with 1 to 5 bacteria, the second shows glands with 6 to 11 bacteria, etc. Glands with zero bacteria were excluded from this analysis. Download [file mbo005163029sf4.tif]
